# The transcription factor ZNF248 promotes colorectal cancer metastasis by binding to ZEB1

**DOI:** 10.7150/jca.92886

**Published:** 2024-08-19

**Authors:** Yanying Ren, Xiaoxu Sun, Xin Chen, Shuai Shao, JingTong Tang, Zhaohui Xu, Yang Xu, Haonan Kang, Liming Wang

**Affiliations:** 1 The Second Hospital of Dalian Medical University, Dalian, Liaoning Province, China.; 2 Dalian Medical University, Dalian, Liaoning Province, China.; 3 Department of Gastrointestinal Surgery, the first Hospital of China Medical University, Shenyang, Liaoning Province, China.

**Keywords:** ZNF248, ZEB1, colorectal cancer, transcription factor, metastasis, epithelial-mesenchymal transition

## Abstract

Colorectal cancer (CRC) is one of the most common malignant tumors globally, with metastasis emerging as the leading cause of mortality in CRC patients. Transcription factors play pivotal roles in the metastatic process. Using bioinformatics tools, we analyzed the TCGA-COAD and GES146587 datasets and identified ZNF248 participating in tumor progression. By analyzing 100 CRC patient tissues, it is found that ZNF248 is highly expressed in cancer tissue as well as in CRC cell lines identified by qRT-PCR. Our study discovered that ZNF248 enhances CRC cell migratory and invasive capabilities. A positive correlation was found between ZNF248 and epithelial-mesenchymal transition (EMT)-related markers (ZEB1, snail1), while E-cadherin exhibited a negative correlation with ZNF248. In addition, the analysis of the TCGA dataset demonstrated a strong correlation between the mRNA level of ZNF248 and ZEB1 expressions. Furthermore, it is found that the overexpression of ZEB1 could reverse CRC cell invasion and migration, along with the inhibition on EMT marker expressions induced by the RNA interference with ZNF248. Immunohistochemical analysis indicated a substantial association of ZNF248 expression with the lymph node metastasis, and with the liver metastasis (P =0.01, P =0.01), and a positive correlation between ZNF248 and ZEB1 expression (P =0.021) was also identified. Using Chip-PCR assay, it is found that ZNF248 bound to the ZEB1 promoter region. These findings showed that ZNF248 promotes CRC metastasis in vivo, revealed its role as an oncogene in CRC by targeting ZEB1 and activating the EMT pathway, which provided novel and promising biomarkers for CRC therapy through targeting ZEB1.

## Introduction

Colorectal cancer (CRC) ranks the third cancer and is one of the leading deaths caused by cancers worldwide 1. Metastasis is the primary cause of the mortalities of CRC patients 2. Tumoral malignancy, including epithelial-mesenchymal transition (EMT) and drug resistance, is crucial in driving tumor metastasis 3. EMT is a process of malignant epithelial cells, in which malignant epithelial cells lose their epithelial characteristics and acquire a mesenchymal phenotype. This transformation is accompanied by the changes of epithelial and mesenchymal-related proteins, such as E-cadherin, beta-catenin, Vimentin, N-cadherin, and Fibronectin 4. EMT promotes CRC cell invasion, migration, and drug resistance, and also alters varieties of signaling pathways.

The transcription factor, ZEB1, plays an important role in the genesis and development of several malignant tumors 5-7. Recent reports showed that ZEB1 can recruit histone deacetylases, DNA methyltransferases, and ubiquitin ligases to reduce E-cadherin expression 8. Therefore, ZEB1 is considered a critical factor functioning in the EMT process.

ZNF248 is located on the human chromosome 10p11.12 and is a member of the C2H2 Zinc finger protein family. This family is characterized by CX2CX3FX5LX2HX3H, which indicates a zinc finger structure containing zinc, cysteine, and histidine residues. This motif consists of two β sheets and a spiral structure 9, which suggests C2H2 is vital for DNA transcription and protein synthesis 10-12.

This study was to identify the effects of ZNF248 on the expression of ZEB1. First, we found a positive association between ZNF248 and the poor survival of CRC patients in the TCGA dataset and GES146587 dataset. Second, Correlation Analysis using GEPIA demonstrated a positive correlation between ZEB1 and ZNF248 in COAD and READ tumors. Finally, we found that ZNF248 may function as a transcription factor to regulate ZEB1 expression through the Cistrome Data Browser. There is no prior research on ZNF248 and its role in CRC. In this study, we assessed ZNF248 and ZEB1 expressions, and Chromatin Immunoprecipitation (ChIP) experiments were employed to confirm that ZNF248 is a transcription factor binding to ZEB1 promoter. Moreover, through in vivo and in vitro experiments, we found that ZNF248 promoted the EMT-related CRC malignancy. In conclusion, ZNF248 may be an effective therapeutic target for the inhibition of CRC metastasis.

## Materials and methods

### Network database analysis

The gene expressions in both cancer and normal tissue within the GSE146587 dataset through GEO database (https://www.ncbi.nlm.nih.gov/geo/). GEPIA was employed to estimate survival differences using data from the TCGA and GEPIA databases 13. Finally, the samples were categorized into low and high-expression groups based on the median level of ZNF248 expression using TCGA (http://cancergenome.nih.gov) database. GSEA was performed for pathway enrichment analysis.

### Tissue samples and cell lines

This study was approved from the academic review board of the Second Hospital of DaLian Medical University. One hundred fresh pairs of CRC cells and adjacent tissues were obtained from the Second Hospital of DaLian Medical University. All specimens were pathologically diagnosed with colorectal cancer, and clinical data were staged according to the eighth edition of UICC. Inclusion of patients: 1. Patients with a histologically confirmed diagnosis of colorectal cancer. 2. Specimens from patients who have not yet received any treatment (chemotherapy, radiation, or surgical intervention). 3. The patients ranged in age from 18 to 80. 4. The patients signed informed consent. Exclusion of patients: Patients with a history of other malignancies.” Each resident patient signed an approval and consent form. HCT116 and SW480 human CRC cell lines were purchased from the Cell Bank of the Chinese Academy of Sciences (Shanghai, China) and maintained in recommended growth media supplemented with 10% fetal calf serum (Gibco Invitrogen, CA).

SW480 is a cell line commonly used in biomedical research, particularly in cancer studies. It originates from as human colorectal adenocarcinoma and is known for its aggressive behavior and resistance to chemotherapy. HCT116 is a cell line derived from human colorectal carcinoma and widely utilized for studying various aspects of cancer biology, including tumor growth, invasion and response to treatment.

### RNA extraction and Quantitative Real-Time PCR

Total RNA was extracted from fresh CRC cell lines and colorectal tissue stored in liquid nitrogen using Trizol (Takara, JAPAN). ZNF248 and GAPDH levels were measured using a NanoDrop ND-100 instrument (NanoDrop, USA), and reverse transcription and amplification were carried out using a Takara kit. Thermocycling conditions were as follows: 95° for 30 s, and then 45 cycles of 95° for 5 s and 60° for 34 s. The following primers were used: ZNF248 forward primer: 5ʹ-TCACCAGGATCTCAGTCAGCCAAG-3ʹ; reverse primer: 5ʹ-CTGCCTCATCATGGAAGCCTTGTC-3ʹ; GAPDH forward primer: 5ʹ-CATGAGAAGTATGACAACAGCCT-3ʹ; reverse primer: 5ʹ- AGTCCTTCCACGATACCAAAGT-3ʹ.

### Protein isolation and western blot

Total proteins were extracted from CRC cell lines using RIPA lysate containing 1% PSMF. Protein samples were separated on 10% sodium dodecyl sulfate-polyacrylamide gels and then transferred protein to PVDF membranes. PVDF membranes were blocked by 5% nonfat milk for 2 h and subsequently incubated with Rabbit ZNF-248 antibody at 1:1000 dilution (ZEN-BIOSCIENCE, China), Rabbit E-cadherin antibody at 1:1000 dilution (Proteintech, USA), Rabbit ZEB1 antibody at 1:1000 dilution (Proteintech, USA), Rabbit SNAIL1 antibody at a 1:500 dilution (Proteintech, USA), and Mouse GAPDH antibody at 1:3000 dilution (Proteintech, USA). PVDF membranes were incubated the next day with corresponding secondary antibodies (Proteintech, USA) for 2 h. Each experiment was repeated three times.

### Immunohistochemistry (IHC)

Paraffin-embedded CRC tissue sections (4 μm thick) were deparaffinized at 65 °C for 2 h, washed with xylene and ethanol, and subjected to high-pressure antigen repair for 3 min. Sections were treated with 3% hydrogen peroxide and 10% normal goat serum for 15 min each, followed by incubation with Rabbit ZNF-248 antibody at a 1:100 dilution (ZEN-BIOSCIENCE, China) and Rabbit ZEB1 antibody at a 1:200 dilution (Proteintech, USA) at 4 °C overnight. Sections were incubated with secondary antibodies, treated with streptavidin-peroxidase reagent, stained with DAB, counterstained with hematoxylin and observed under a microscope. Immunohistochemical scoring was based on staining intensity (scored as 0-3: negative, weak, medium, and strong) and the extent of staining (scored as 0: < 5%, 1: 5%-25%, 2: 26%-50%, 3: 51%-75%, and 4: > 75%) relative to the whole carcinoma.

### RNA interference

ZNF248 siRNA and negative control (NC) were designed by GenePharma (China) with the following sequences: si-1 Sense: GGGAGCUUCUAUUCCACAATT; Anti-sense: UUGUGGAAUAGAAGCUCCCTT; si-2 Sense: GCCUGAUGAGUUUAAUGUATT; Anti-sense: UACAUUAAACUCAUCAGGCTT; Negative control Sense: UUCUCCGAACGUGUCACGUTT; Anti-sense: ACGUGACACGUUCGGAGAATT.

HCT116 and SW480 CRC cells were transfected with ZNF248 si1, ZNF248 si2, NC, and lipo3000 (Invitrogen, USA) for 48 h following the instructions. Western blot was utilized to assess the inhibitory effect.

### Immunoprecipitation

Lysate, containing 20 mM Tris/HCl (pH7.4), 1.0% NP- 40, 150 mM NaCl, 1 mM EDTA, 10 μg/mL albumin, and 50 μg/mL PMSF, was used to extract total protein samples from CRC cell lines. Rabbit anti-ZNF248 (ZEN-BIOSCIENCE, China) at a 1:100 dilution and Rabbit anti-IgG (CST, USA) at a 1:100 dilution was mixed with magnetic beads and incubated at 4 °C for 4 h. CRC cell proteins were then added to the antibody and magnetic beads complex, which was incubated at 4 °C overnight. The next day, immunoprecipitation samples separated from magnetic beads were analyzed by Western Blot.

### Chromatin immunoprecipitation

CRC cells grown to 90% confluence in a 15 cm cell culture dish and then cross-linked with fresh formaldehyde. Glycine was added to terminate the cross-linking process. Complexes were collected in PBS containing protease inhibitors. Following the SimpleChIP ® Plus Enzymatic Chromatin IP Kit (CST, USA) instructions, 10 μl Rabbit anti-ZNF248 (ZEN-BIOSCIENCE, China), 2 μL Rabbit anti-IgG were used to extract target DNA. Primers were designed according to potential binding sites, and DNA was amplified using a Takara kit (JAPAN).

### Wound healing assay

Transfected HCT116 and SW480 cells were placed in a 6-well pallet. Once the cell fusion reached 80% confluence, a gentle scrape was made on the bottom of the well using a 200 μL pipette tip. The was washed with PBS three times, and images were acquired at 0 and 24 h using a microscope.

### Invasion and migration assay

Transfected 3*10^4^ HCT116 and 6*10^4^ SW480 cells suspended in 300 μL of serum-free medium were placed in the upper chamber, while 600 μL of 10% FBS medium was added to the lower chamber. After 24 h, the chambers were fixed in ice-cold methanol and stained with crystal violet (Sigma, USA) for 30 min. Migratory cells were then captured in four random fields per well (Nikon, Japan). Each experiment was repeated three times.

### Mice xenograft model

The Ethics Committee of DaLian Medical University approved mice experiments. For the liver metastasis model, 6-week-old non-SCID mice were obtained from Cyagen Bioscience (China). A small incision was made under the left abdominal flank to expose the spleen. Transfected CRC cells (1*10^6^) suspended in 100 μL pre-cold PBS were injected into the inferior pole of the spleen. The mice were sacrificed, and liver tissue was obtained one month later. Tumor size was measured using vernier calipers, and liver metastasis tumors were detected by hematoxylin and eosin (HE) staining.

### Statistical analysis

Results from the three independent experiments were presented as the mean ± standard deviation. The Wilcoxon rank-sum test analysis was used to estimate differences between the two groups. Correlation analysis was performed using the chi-square test. Survival was evaluated using the Kaplan-Meier method, and differences were analyzed using the log-rank test. All statistical analyses were performed by SPSS 17.0 (Chicago, IL, USA), and P<0.05 was considered statistically significant.

## Results

### Overexpression of ZNF248 predicts poor survival in CRC patients

**Figure 1:**
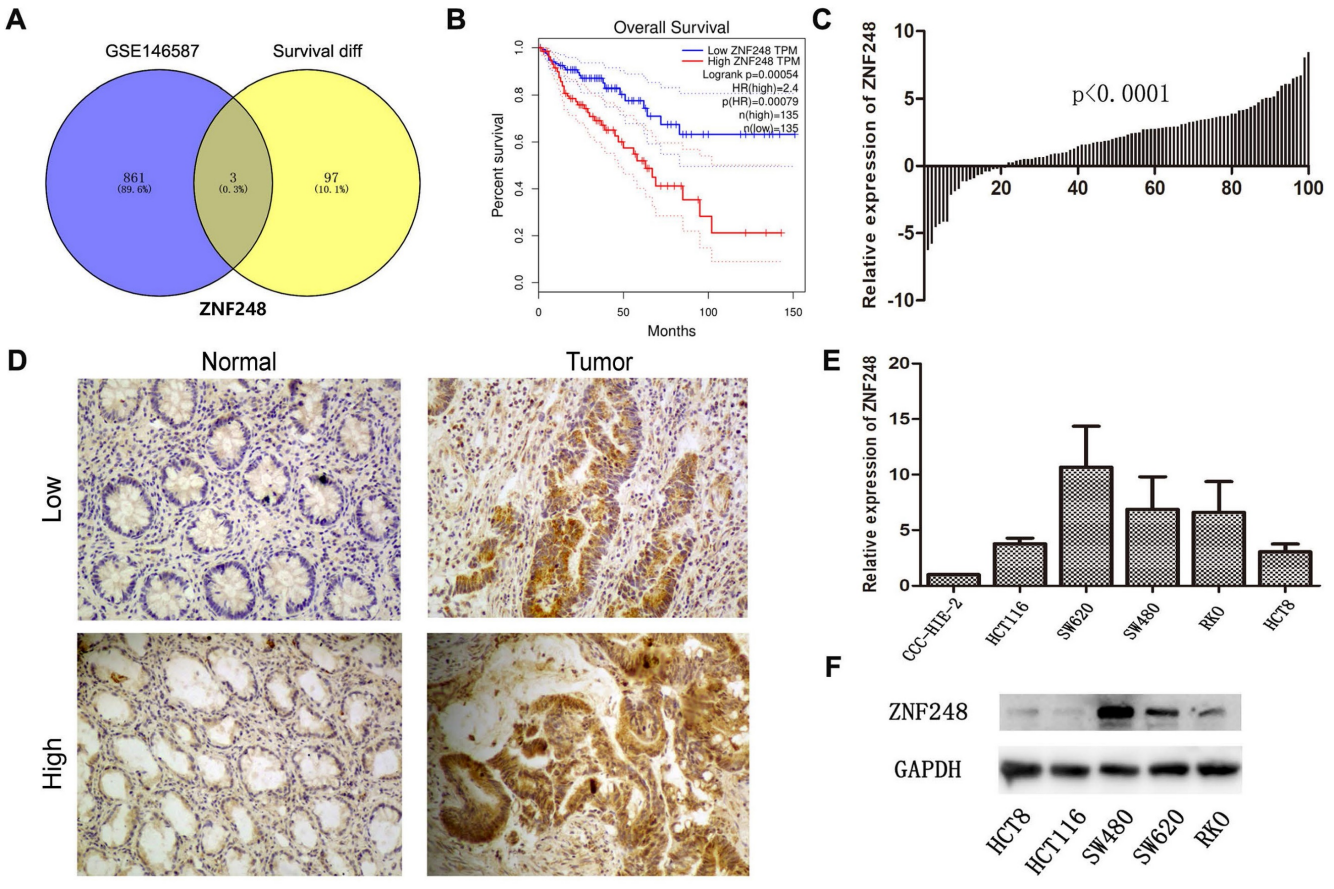
ZNF248 expressions in CRC tissues and cells. (A) The same intersection genes between differential genes in the GSE146857 dataset and survival-related genes in the TCGA database by Venn diagram. (B) High and low ZNF248 expressions plotted against overall survival time in 270 CRC patients in the TCGA database. (C) mRNA transcripts of ZNF248 in 100 pairs of CRC tissues and adjacent tissues by qRT-PCR. (D) Protein expressions of ZNF248 in 100 pairs of CRC tissues and adjacent tissues by IHC. (E) mRNA transcripts of ZNF248 in 5 CRC cell lines and one normal intestinal epithelial cell qRT-PCR. (F) Protein expressions of ZNF248 in 5 CRC cell lines and one normal intestinal epithelial cell by WB. *, P<0.05; **<0.01 compared with the control.

To identify novel genes that are associated with CRC, the relationship between mRNA expression and clinical information of CRC patients and normal individuals from TCGA and GTEx datasets was comprehensively examined. Among 100 genes, the mRNA expression levels were found to be significantly related to patients' survival. Additionally, in the GSE146587 dataset, 864 genes were identified to show different mRNA expression levels in cancer tissue compared to adjacent tissue. ZNF248 was identified as one of the top-ranked genes using a Venn diagram (Figure 1A). On the other hand, the expression of ZNF248 in CRC tissues was evaluated using IHC and qRT-PCR assay. Consistent with bioinformatic data, a high expression level of ZNF248 was observed in cancer tissue (Figure 1B, C). ZNF248 expression among various human cancers was next assessed. As demonstrated in Table 1, ZNF248 expression is significantly associated with node metastasis and liver metastasis (p=0.01, p=0.01). Furthermore, the mRNA expression of ZNF248 in normal intestinal epithelial cell line and five CRC cell lines was determined by qPCR. The results indicated that ZNF248 is highly expressed in RKO and SW480 but not in HCT116. Therefore, SW480 and HCT116 were selected for further experiments (Figure 1D, E). Taken together, ZNF248 overexpression is an adverse factor for CRC patients.

### ZNF248 promoted metastatic ability of CRC cells

**Figure 2:**
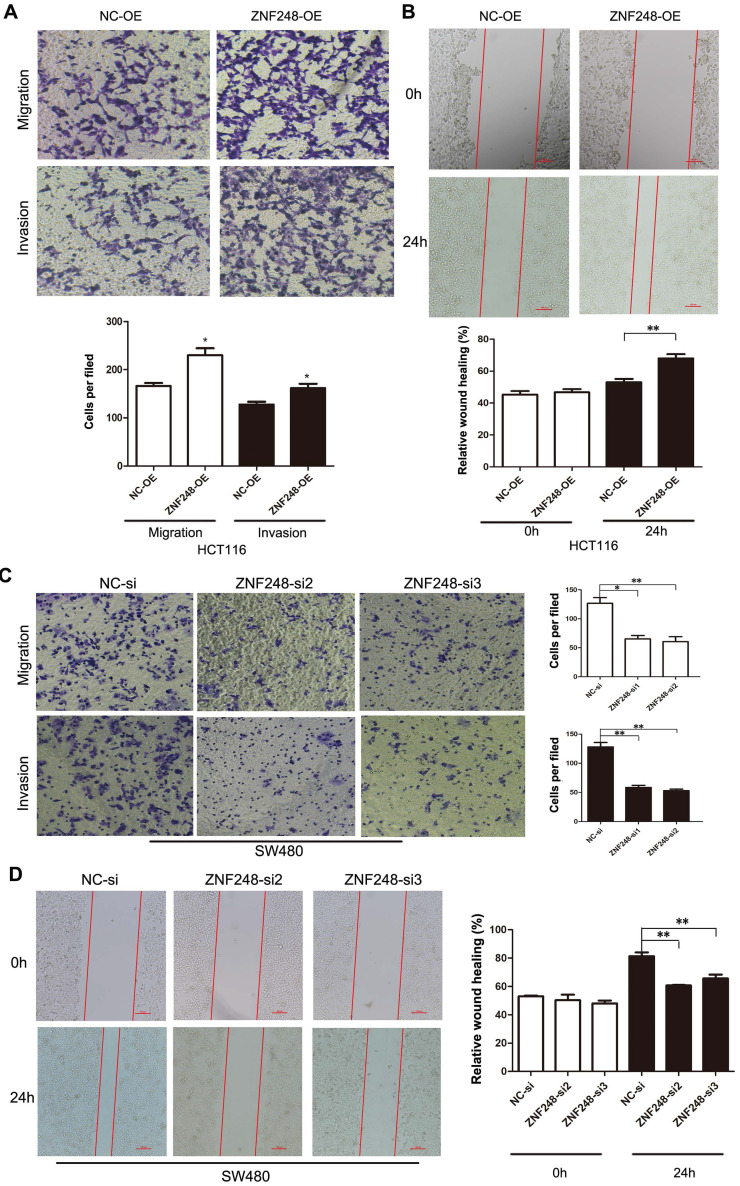
ZNF248 promoted CRC cell invasion and migration. (A) Cell migration and invasion in HCT116 transfected ZNF248 plasmid. (B) Wound healing in HCT116 transfected ZNF248 plasmid. (C) Cell migration and invasion in SW480 transfected ZNF248 siRNA. (D) Wound healing in SW480 transfected ZNF248 siRNA. Bars indicate Mean ± SE, *P<0.05; **p<0.01 (X20 magnification), n=3.

The effect of ZNF248 on migration and invasion ability of CRC cells was investigated using transwell assays and wound healing experiments. The results showed that the ectopic expression of ZNF248 significantly promoted CRC cell migration and invasive capability (Figure 2A, B). In contrast, silencing of ZNF248 reduced CRC cell invasion and migration, and delayed wound healing in SW480 cells (Figure 2C, D). These data suggest that ZNF248 promotes metastatic ability of colorectal tumor cells.

### ZNF248 silencing inhibited EMT-related markers in CRC

**Figure 3:**
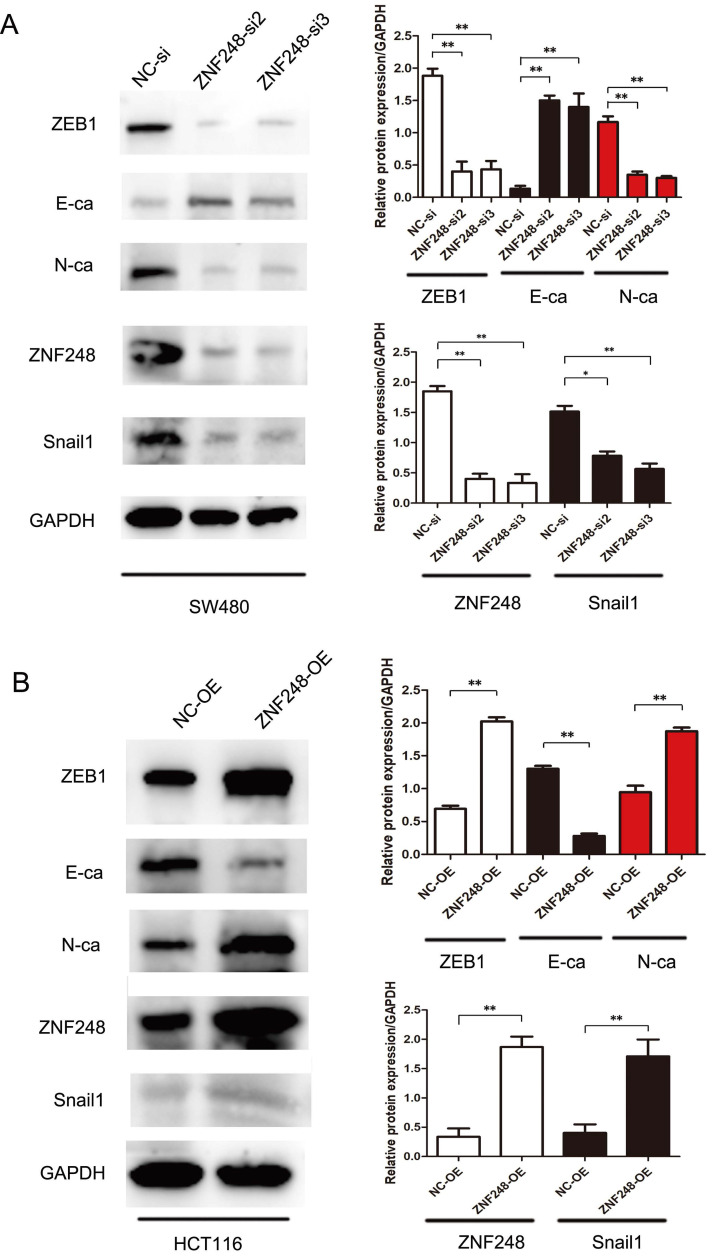
ZNF248 regulates ZEB1 and EMT-related markers. (A) The protein expressions involving ZEB1, SNAIL1, E-cadherin, and other EMT-related markers in ZNF248 siRNA and negative control transfected SW480. (B) The protein expressions involving ZEB1, SNAIL1, E-cadherin, and other EMT-related markers in ZNF248 overexpression plasmid and negative control transfected HCT116. Bars indicate Mean ± S.E.*, P<0.05; **, P<0.01 compared with the control, n=3.

To gain insights into the molecular mechanisms underlying the pro-tumorigenic action of ZNF248, several important cancer pathways were screened using GSEA analysis. The results showed that ZNF248 is related to the cell adhesion signaling pathway (Supplementary Figure 1). ZEB1, a transcription factor of EMT, is proved to regulate cell adhesion 14-15. In order to confirm whether the function of ZNF248 depends on ZEB1 activation, CRC cells with or without ectopic expression of ZNF248 were treated with two different cell lines. Specifically, ZNF248 expression was inhibited in SW480 cells and increased in HCT116 cells. When ZNF248 was silenced in SW480, the expressions of ZEB1 and SNAIL1 decreased, whereas E-cadherin expression increased (Figure 3A). In contrast, ZNF248 overexpression decreased the EMT expressions of ZEB1, SNAIL1, and E-cadherin in HCT116 cells (Figure 3B). Furthermore, ZNF248 was found to bind to ZEB1 in two colon cancer cells using IP assays (Figure 5A). Consistent result was observed in the co-expression analysis, which showed a close association between ZNF248 and ZEB1 in the cytoplasm by IHC (Figure 5B, Table 2). Correlation analysis also showed that ZNF248 mRNA was positively associated with ZEB1 mRNA in GEPIA (Figure 5C). Thus, these data suggest that ZNF248 silencing may inhibit EMT-related markers in CRC.

### ZNF248 regulates the process of EMT by binding to the ZEB1 promoter region in CRC cells

**Figure 4:**
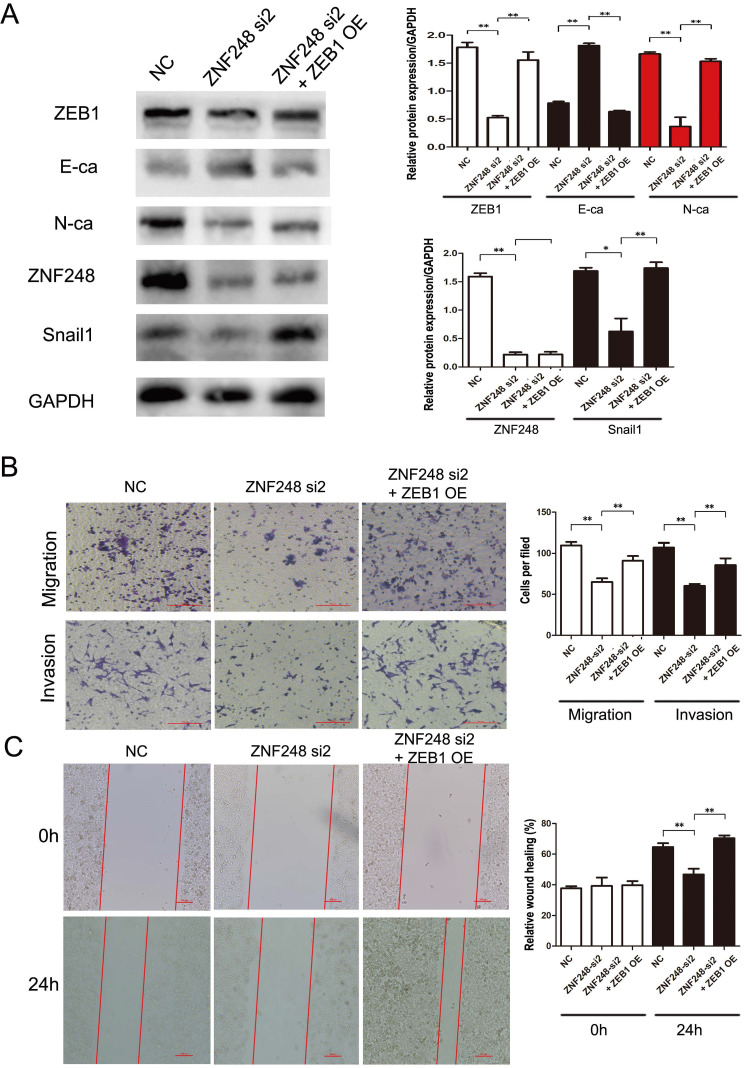
ZEB1 overexpression rescues the effect of ZNF248 silencing in CRC cell lines. (A) ZEB1, SNAIL1, and E-cadherin changes in rescue experiments. (B) Cell invasion and migration in rescue experiment. (C) Wound healing in rescue experiment. Bars indicate Mean ± S.E.*, P<0.05; **, P<0.01 compared with the control, n=3.

**Figure 5:**
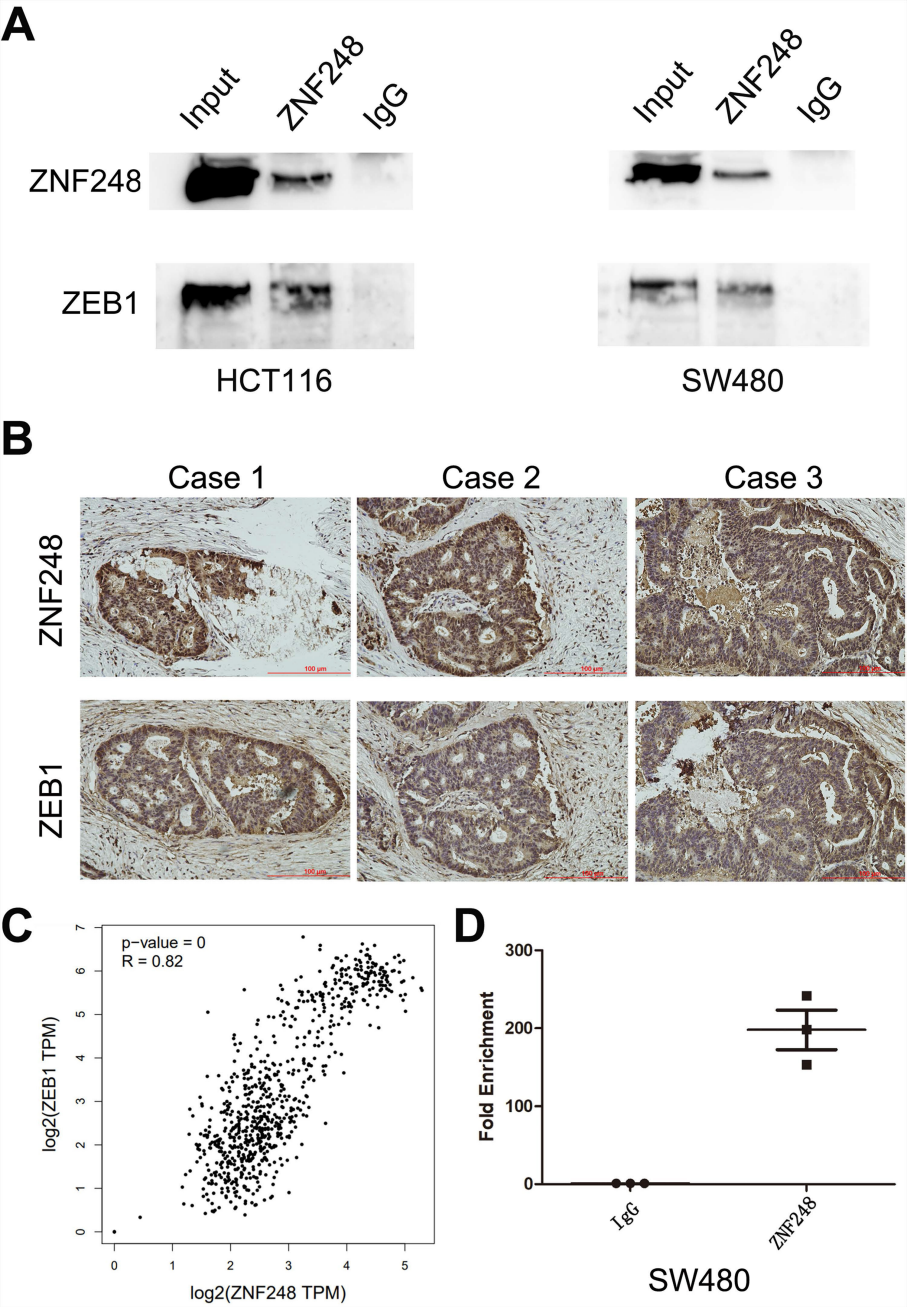
ZNF248 acts as a transcription factor of ZEB1. (A) ZEB1 co-immunoprecipitated with ZNF248 in CRC cells. The input and IgG lanes were used as the positive and negative controls, respectively. (B) Co-expression of ZNF248 with ZEB1 in CRC tissues using Immunohistochemistry, p=0.021. (C) mRNA Co-expression of ZNF248 with ZEB1 in TCGA and GTEx database by GEPIA analysis. (D) ZNF248 is a transcription factor binding ZEB1 promoter region by Chip-PCR in SW480. Bars indicate Mean ± S.E.*, P<0.05; **, P<0.01 compared with the control, n=3.

ZNF248 is identified as a member of zinc transcription factor family. To further explore the molecular mechanism of ZNF248 in CRC cells, ZNF248-related Chip-seq data were analyzed on the Cistrome Data website and compared with those of UCSC website (Supplementary Figure 2A). Peak enrichment was observed in the ZEB1 promoter region. Subsequently, primers for Chip-PCR were designed based on these peaks. ZNF248 was identified as a transcription factor of ZEB1 by Chip-PCR (Figure 5D). Further verification was performed using agarose gel electrophoresis, which indicated that ZNF248 is indeed the transcription factor of ZEB1 (Supplementary Figure 2B). Meanwhile, Double luciferase reporter gene assay showed that ZNF248 regulate ZEB1 transcription (p = 0.002) (Supplementary Figure 2C). Overexpression of ZEB1 restored the changes in invasion and migration ability and the expression of ZEB1, SNAIL1 and E-cadherin. Rescue experiment further supports the conclusion of this study (Figure 4A, B, C).

### ZNF248 silencing inhibited CRC tumors in liver metastasis in vivo

**Figure 6:**
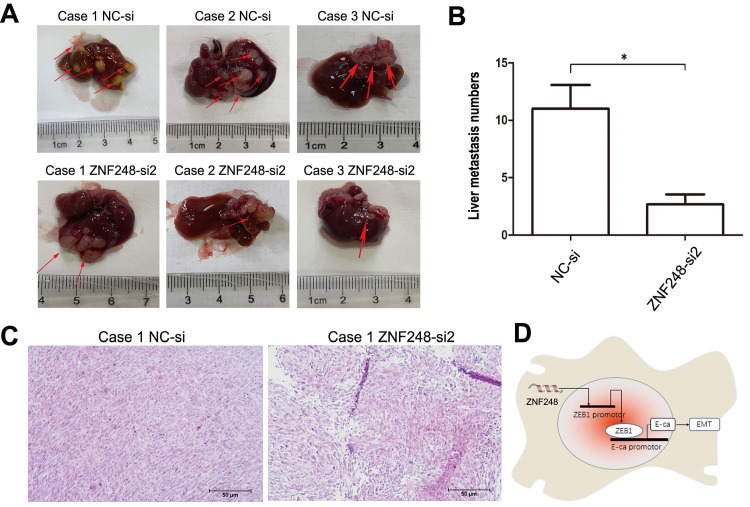
ZNF248 interfering inhibited liver metastasis in vivo. (A) Non-SCID mice liver metastasis model in NC and ZNF248 siRNA groups. (B) The numbers of liver metastasis in NC and ZNF248 siRNA groups. (C) Metastatic tumors were identified by HE staining. (D) The diagram illustrates how ZNF248 regulated the expression of ZEB1 and the EMT process in CRC. Bars indicate mean ± SE, * P <0.05, **p<0,01, comparing with NC, n=3.

To further investigate the tumourigenic ability of ZNF248, SW480 cells with silenced ZNF248 were injected into the spleens of Nod-SCID mice. As shown in Fig. 6A, C, metastatic tumors were identified by HE staining. The numbers and sizes of liver metastasis of ZNF248 siRNA group was decreased as compared with NC group (Figure 6B). Xenograft model experiments further verified that ZNF248 was important in CRC liver metastasis. Collectively, these data imply that ZNF248 plays a pivotal role in promoting the mesenchymal-epithelial transition of colorectal cancer, ultimately regulating the occurrence of liver metastasis in CRC (Figure 6D).

## Discussion

The ZNF transcription factor family plays a crucial role in the progression of various types of tumors. ZNF248, a member of the ZNF transcription factor family encoding a C2H2 type transcription factor, has not been studied in CRC. In this study, the bioinformatics analysis revealed that ZNF248 is highly expressed in CRC tissue, positively correlating with poor patient survival. Both qRT-PCR and IHC results confirmed that ZNF248 expression was elevated in cancer tissues, closely associated with lymph nodes and distant metastasis (p=0.01).

The high mortality of colorectal cancer is attributed to the invasion and migration of CRC cells. Despite undergoing chemoradiotherapy, some patients still developed distant metastasis 16. Therefore, the current focus is to understand the mechanisms of CRC migration and invasion. EMT is characterized by the loss of polarity in malignant cells derived from epithelial tissues, accompanied by the disruption of cell-cell and cell-extracellular adhesion, and reorganization of the cytoskeleton. Thus, EMT could promote CRC cell invasion and metastasis 17-19. EMT has been widely considered to be important in the metastasis and recurrence of various tumors 20-22. Here, our findings showed that ZNF248 plays a significant role in CRC development and is closely linked to lymph nodes and distant metastasis. Apart from promoting CRC cell growth, ZNF248 promoted CRC cell migration and invasion. Western blot analysis also showed that ZNF248 regulated the EMT process. In accordance with *in vitro* data, the non-SCID mice liver metastasis model further validated the crucial role of ZNF248 in the EMT process of CRC *in vivo*. Hence, the remarkable oncogenic effect in CRC cells *in vitro* and* in vivo* suggests that ZNF248 play an important role in CRC progression.

The current study focuses on unraveling the specific mechanisms by which ZNF248 regulates EMT. TGF-β, PI3K-AKT, RAS-ERK1/2, and Wnt-β-catenin signaling pathways have been implicated in the EMT process of CRC 23-26. Additionally, several transcription factors, such as ZEB1, ZEB2, Snail1, and Slug, are known to regulate E-cadherin, an EMT-related marker 27-30. Among them, ZEB1 is a pivotal transcription factor of E-cadherin. Wu et al. 31 reported that the RP11/hnRNPA2B1/mRNA complex accelerated the mRNA degradation of two E3 ligases, Siah1 and Fbxo45, and subsequently prevented the proteasomal degradation of ZEB1. Based on the bioinformatics analysis, we hypothesized that ZNF248 may be the transcription factor of ZEB1. Meanwhile, our findings revealed that ZNF248 increases the expression of ZEB1, inhibits E-cadherin expression and promotes CRC cell invasion and migration. In agreement with the results, rescue experiments showed that up-regulation of ZEB1 restored the impaired invasion and migration abilities of CRC cells caused by ZNF248 down-regulation. Finally, ChIP experiments demonstrated that ZNF248 enhanced the EMT process of CRC cells by binding to the ZEB1 promoter region.

In summary, we have demonstrated for the first time that ZNF248 plays a pivotal role in the malignant biological behavior of CRC. The ZNF248-ZEB1 signaling pathway promotes EMT in CRC cells. Importantly, ZNF248 emerges as a novel marker for CRC patients' survival and treatment outcomes.

## Supplementary Material

Supplementary figures and table.

## Figures and Tables

**Table 1:** The relationship between ZNF248 and 100 CRC patients' clinical pathology data.

		Expression of ZNF248		P value
Parameters	No. of patients	Low	High	
**Age(years)**				0.29
<60	37	12	25	
≥60	63	28	35	
**Gender**				0.84
Male	57	20	37	
Female	43	20	23	
**Size (maximal diameter)**				
≥5cm	37	16	21	
<5cm	63	24	39	
**Differentiation**				
Well, Moderate	80	31	49	
Poor	20	9	11	
**TNM stage**				0.35
Ⅰ+Ⅱ	62	30	32	
Ⅲ+Ⅳ	38	10	28	
**Tumor invasion**				
T1+T2+T3	72	30	42	
T4	28	10	18	
**Lymph node status**				**0.01**
Positive	59	18	41	
Negative	41	22	19	
**Metastasis**				**0.01**
M0	77	35	42	
M1	23	5	18	

**Table 2:** The association of ZNF248 and ZEB1 in 40 CRC clinical samples.

		ZNF248			
		High	Low	Pearson	P value
**ZEB1**	High	24	3	0.393	0.021
	Low	7	6		

## References

[1] Siegel RL, Miller KD, Jemal A (2019). Cancer statistics, 2019. CA Cancer J Clin.

[2] Jemal A, Siegel R, Ward E (2008). Cancer statistics, 2008. CA Cancer J Clin.

[3] Vu T, Datta PK (2017). Regulation of EMT in Colorectal Cancer: A Culprit in Metastasis. Cancers (Basel).

[4] Cao H, Xu E, Liu H (2015). Epithelial-mesenchymal transition in colorectal cancer metastasis: a system review. Pathol Res Pract.

[5] Aghdassi Ali, Sendler et al (2012). Recruitment of histone deacetylases HDAC1 and HDAC2 by the transcriptional repressor ZEB1 downregulates E-cadherin expression in pancreatic cancer. Gut.

[6] Yang Y, Ahn YH, Chen Y (2014). ZEB1 sensitizes lung adenocarcinoma to metastasis suppression by PI3K antagonism. J Clin Invest.

[7] Wang Y, F Bu, Royer C (2014). ASPP2 controls epithelial plasticity and inhibits metastasis through β-catenin-dependent regulation of ZEB1. Nat Cell Bio.

[8] Schneider G, Kramer O H, Saur D (2012). A ZEB1-HDAC pathway enters the epithelial to mesenchymal transition world in pancreatic cancer. Gut.

[9] Nunez N, Clifton M, Funnell A (2011). The multi-zinc finger protein ZNF217 contacts DNA through a two-finger domain. Journal of Biological Chemistry.

[10] Brayer K J, Kulshreshtha S, Segal D J (2008). The Protein-Binding Potential of C2H2 Zinc Finger Domains. Cell Biochemistry & Biophysics.

[11] Ma H, Ng H M, Teh X (2014). Zfp322a Regulates Mouse ES Cell Pluripotency and Enhances Reprogramming Efficiency. PLoS Genetics.

[12] Lehmann W, D Mossmann, Kleemann J (2016). ZEB1 turns into a transcriptional activator by interacting with YAP1 in aggressive cancer types. Nat Commun.

[13] Tang Z, Li C, Kang B (2017). GEPIA: a web server for cancer and normal gene expression profiling and interactive analyses. Nucleic Acids Res.

[14] Liu M, Zhang Y, Yang J (2021). Zinc-Dependent Regulation of ZEB1 and YAP1 Coactivation Promotes Epithelial-Mesenchymal Transition Plasticity and Metastasis in Pancreatic Cancer. Gastroenterology.

[15] Al-Hajj M, Wicha MS, Benito-Hernandez A (2003). Prospective identification of tumorigenic breast cancer cells. Proc Natl Acad Sci.

[16] House MG, Kemeny NE, Gönen M (2011). Comparison of adjuvant systemic chemotherapy with or without hepatic arterial infusional chemotherapy after hepatic resection for metastatic colorectal cancer. Ann Surg.

[17] M (2009). Yilmaz, G. Christofori. EMT, the cytoskeleton, and cancer cell invasion. Cancer Metastasis Rev.

[18] Tang J, Gao W, Liu G (2021). miR-944 Suppresses EGF-Induced EMT in Colorectal Cancer Cells by Directly Targeting GATA6. Onco Targets Ther.

[19] Sheng W, Chen C, Dong M (2017). Calreticulin promotes EGF-induced EMT in pancreatic cancer cells via Integrin/EGFR-ERK/MAPK signaling pathway. Cell Death Dis.

[20] Tang Q, Chen J, Di Z (2020). TM4SF1 promotes EMT and cancer stemness via the Wnt/β-catenin/SOX2 pathway in colorectal cancer. J Exp Clin Cancer Res.

[21] Ombrato L, Nolan E, Kurelac I (2019). Metastatic-niche labelling reveals parenchymal cells with stem features. Nature.

[22] Shapiro IM, Cheng AW, Flytzanis NC (2011). An EMT-driven alternative splicing program occurs in human breast cancer and modulates cellular phenotype. PLoS Genet.

[23] Wang X, Lai Q, He J (2019). LncRNA SNHG6 promotes proliferation, invasion and migration in colorectal cancer cells by activating TGF-β/Smad signaling pathway via targeting UPF1 and inducing EMT via regulation of ZEB1. Int J Med Sci.

[24] Wei R, Xiao Y, Song Y (2019). FAT4 regulates the EMT and autophagy in colorectal cancer cells in part via the PI3K-AKT signaling axis. J Exp Clin Cancer Res.

[25] Zhao J, Ou B, Han D (2017). Tumor-derived CXCL5 promotes human colorectal cancer metastasis through activation of the ERK/Elk-1/Snail and AKT/GSK3β/β-catenin pathways. Mol Cancer.

[26] Li Q, Lai Q, He C (2019). RUNX1 promotes tumour metastasis by activating the Wnt/β-catenin signalling pathway and EMT in colorectal cancer. J Exp Clin Cancer Res.

[27] Ji L, Li X, Zhou Z, Zheng Z, Jin L (2020). LINC01413/hnRNP-K/ZEB1 Axis Accelerates Cell Proliferation and EMT in Colorectal Cancer via Inducing YAP1/TAZ1 Translocation. Mol Ther Nucleic Acids.

[28] Sreekumar R, Al-Saihati H, Emaduddin M (2021). The ZEB2-dependent EMT transcriptional programme drives therapy resistance by activating nucleotide excision repair genes ERCC1 and ERCC4 in colorectal cancer. Mol Oncol.

[29] Freihen V, Rönsch K, Mastroianni J (2020). SNAIL1 employs β-Catenin-LEF1 complexes to control colorectal cancer cell invasion and proliferation. Int J Cancer.

[30] Yao C, Su L, Shan J (2016). IGF/STAT3/NANOG/Slug Signaling Axis Simultaneously Controls Epithelial-Mesenchymal Transition and Stemness Maintenance in Colorectal Cancer. Stem Cells.

[31] Wu Y, Yang X, Chen Z (2019). m^6^A-induced lncRNA RP11 triggers the dissemination of colorectal cancer cells via upregulation of Zeb1. Mol Cancer.

