# Supplementary material for: The transcription factor ZNF248 promotes colorectal cancer metastasis by binding to ZEB1
Source: J Cancer. 2024 Aug 19;15(16):5440–50. doi: 10.7150/jca.92886 (PMC11375549; doi:10.7150/jca.92886)
Supplement: Supplementary file 1 — Supplementary figures and table. [file jcav15p5440s1.pdf]

Supplementary Materials

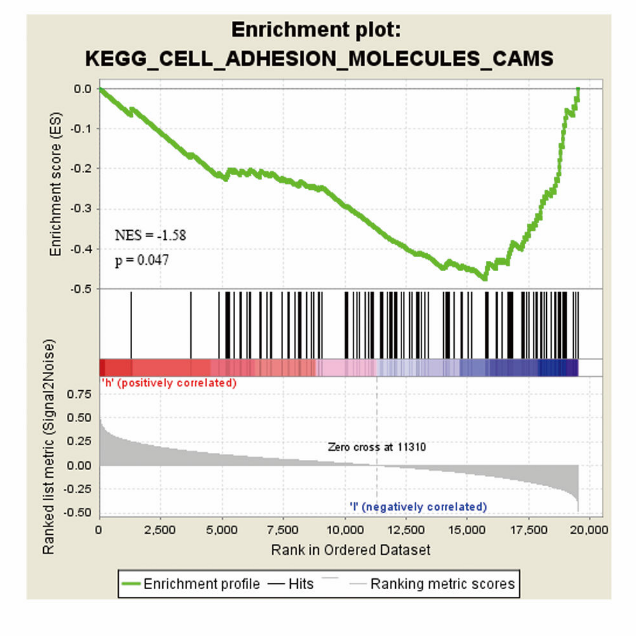

Supplementary Figure 1. ZNF248 is negatively associated with cell adhesion molecules and signaling pathways.

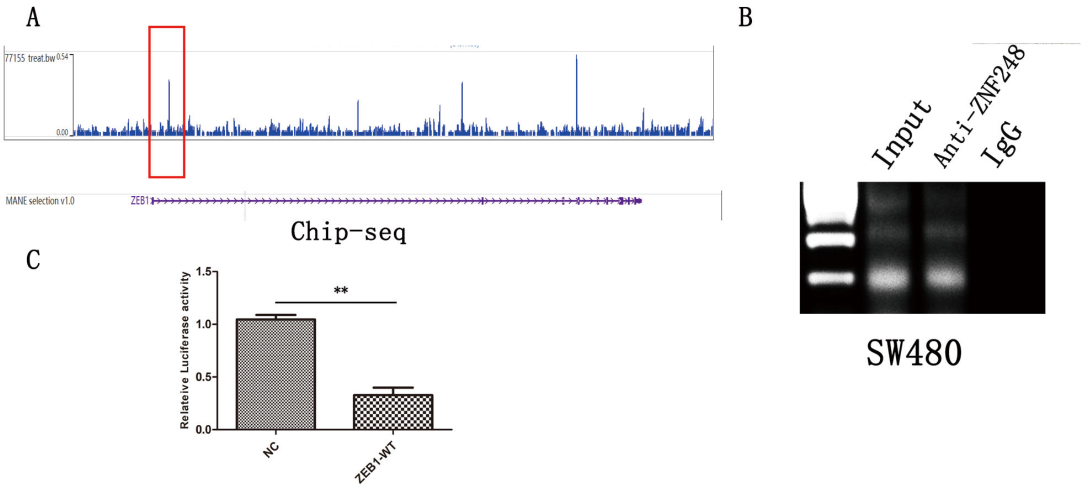

Supplementary Figure 2. ZNF248 binding ZEB1 promoter (A) ZNF248 related Chip-seq data in Cistrome Data website and binding site shown in UCSC. (B) Agarose gel electrophoresis demonstrated the enrichment of ZEB1 using ZNF248 by Chip-PCR. (C) Double luciferase reporter gene assay showed that ZNF248 regulate ZEB1 transcription.

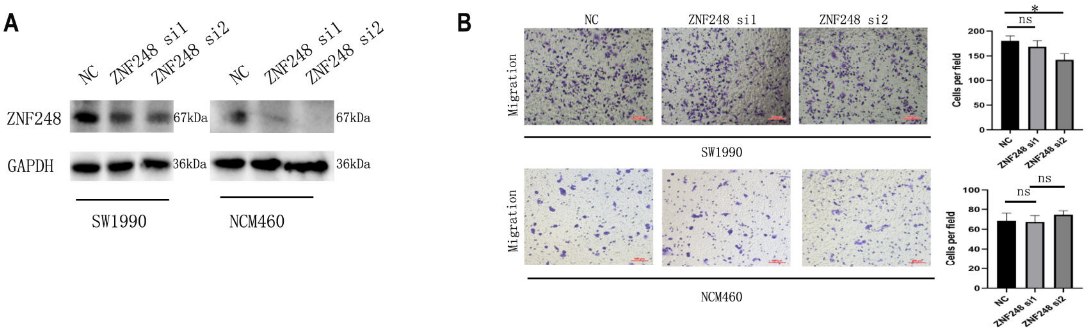

Supplementary Figure 3. Silencing ZNF248 in NCM460 and SW1990.

Supplementary Table 1

The relationship between ZNF248 and TCGA-COAD patients' clinical pathology data

		Expression of ZNF248		*P* value
Parameters	No. of patients	Low (254)	High (256)	
Age(years)	510	68.2 ± 13.57	66.3±12.49	0.10
Gender				0.48
Male	266	137	129	
Femal	244	117	127	
Race				0.61
American	2	0	2	
Asian	11	6	5	
Black	65	20	45	
White	241	116	125	
NA	191	112	79	
T stage				0.61
T1	11	5	6	
T2	86	47	39	
T3	349	174	175	
T4	64	28	36	
Lymph node status				0.31
positive	205	96	109	
negative	305	158	147	
Metastasis				0.02
M0	370	198	172	
M1	72	31	41	
NA	68	25	43	
